# Supplementary material for: Determination of diagnostic cycle threshold cutoffs for qPCR-based prevalence surveys of soil-transmitted helminth infections
Source: Microbiol Spectr. 2026 Sep 2;14(10):e00363-26. doi: 10.1128/spectrum.00363-26 (PMC13689095; doi:10.1128/spectrum.00363-26)
Supplement: Supplementary file 1 [file spectrum.00363-26-s0002.docx]

**Supplementary information**

**S1 Table: Egg spiking experimental approaches to determine qPCR Ct Cut-off for soil-transmitted helminth infections.**

| **Experiment** | **Methods** | | **N** | ***A. lumbricoides* n (%)** |
| --- | --- | --- | --- | --- |
| Experiment 1 | Bead beating, 400 µl lysate DNA extraction | Lyoplate PCR^*^ | 35 | 15 (42.9) |
|  |  | Liquid PCR^**^ |  | 13 (37.1) |
|  | Remaining 400 µl lysate DNA extraction and liquid PCR | |  | 12 (34.3) |
| Experiment 2 | Bead beating, 800 µl lysate (2 aliquots of 400 µL extracted and DNA pooled) and liquid PCR | | 20 | 6 (30.0) |
| Experiment 3 | Increased bead beating from 2 to 12 rounds and liquid PCR | | 15 | 10 (66.7) |
|  | Results from all experiments | | 70 | 31 (45.6^#^) |

Footnote: ^*^Lyoplate PCR: 384-well lyophilized qPCR used in Deworm3 trial; ^**^Liquid PCR: standard non-lyophilized liquid qPCR reagents; ^#^Percentage was calculated for 68/70 replicates as 2 samples failed internal control

**S2 Table: Genome equivalent spiking experiment derived qPCR Ct Cut-off**

| **Species** | **Replicates tested** | **Mean Ct** | **SD** | **+3 SD Cut-off** |
| --- | --- | --- | --- | --- |
| *N. americanus* | 132 | 26.2 | 0.5 | 27.8 |
| *A. duodenale* | 132 | 33.1 | 2.2 | 39.7 |
| *A. lumbricoides* | 132 | 28.8 | 0.6 | 30.6 |
